# Next-Generation Moses Pulse Modulation: An Evaluation of Whether This Is Hype or Breakthrough

**DOI:** 10.7759/cureus.89298

**Published:** 2025-08-03

**Authors:** Yasir Ashraf, Suliman Ali, Muhannad Zamzami, Asad Abedin

**Affiliations:** 1 Department of Urology, University College London, London, GBR; 2 Department of Otolaryngology, Leicester Royal Infirmary, Leicester, GBR; 3 Department of Urology, Warwick Hospital, Warwick, GBR; 4 Department of Urology, Russell’s Hall Hospital, Dudley, GBR

**Keywords:** holep, moses™ technology, prostate enucleation, pulse modulation, urolithiasis

## Abstract

Next-generation Moses™ technology is a pulse modulation modality of the traditional holmium yttrium-aluminum-garnet (YAG) laser and has been developed for use in both laser lithotripsy and prostate enucleation. In traditional holmium YAG lasers, the energy is delivered in a single continuous pulse, which can be less efficient in terms of stone fragmentation and tissue interaction. Moses technology, on the other hand, uses multiple, shorter pulses within a single laser firing cycle, which makes the energy delivery more controlled and effective. In pre-clinical studies, Moses™ technology has been shown to improve stone fragmentation efficiency in lithotripsy and reduce overall procedure time in prostate enucleation. Given its relatively recent introduction, it is still unclear if use of Moses™ can translate to efficiency and time savings in the operating room. The aim of this narrative review is to summarise the latest evidence for use of Moses™ technology in the treatment of urolithiasis and prostate enucleation.

To compare Moses™ to standard holmium, a literature review was conducted in PubMed and Embase for randomised controlled trials and cohort studies. For laser lithotripsy, outcomes investigated were overall procedure time, fragmentation time, degree of retropulsion, stone-free rate and complications. For prostate enucleation, the outcomes were operative time and haemostasis time.

This review summarises current comparative findings between Moses™ and conventional holmium:YAG laser in the context of lithotripsy and prostate enucleation, highlighting consistent advantages in key operative metrics across both applications.

Current literature evidence suggests Moses™ technology is associated with superior outcomes compared to standard holmium YAG laser in several key parameters determining the overall efficiency. For urolithiasis, Moses™ could offer shorter procedure times and for prostate enucleation, it could reduce the learning curve of HoLEP, enabling it to become an outpatient-based procedure.

## Introduction and background

The holmium YAG (yttrium-aluminum-garnet) laser is a widely used laser in urology, particularly in the treatment of kidney stones, benign prostatic hyperplasia (BPH), and other medical conditions requiring precise tissue ablation. It operates in the infrared spectrum, specifically at a wavelength of 2.1 microns, which is highly absorbed by water. This characteristic makes the holmium YAG laser particularly effective in tissues with high water content, such as kidney stones and prostate tissue. The energy produced by the laser is then delivered via a fibre optic cable. The fibre delivers focused energy to the target tissue, which causes photothermal effects. When the laser light hits a kidney stone or prostate tissue, the energy is absorbed by the water content, resulting in rapid heating, vaporisation, and fragmentation of the target. The heat can also cause coagulation of surrounding tissues, which is especially useful in surgical procedures like holmium laser enucleation of the prostate (HoLEP).

The holmium YAG laser is the gold standard in laser lithotripsy and is the most common energy source for laser prostate enucleation [1]. In urinary tract stone treatment, holmium laser has demonstrated its ability to be safe and efficacious in fragmenting stones of all types and its ability to be used with flexible instruments. In addition, it has demonstrated minimal thermal injury to the ureter [2]. Despite these abilities, the holmium laser can be associated with the retropulsion of stones, leading to longer total operating time. Advances in laser technology have now allowed for the modulation of the distribution of pulse energy depending on its purpose.

The holmium YAG laser itself is a solid, pulsed laser emitting light at 2100 nm, its active component being the element holmium, which is combined with an YAG crystal (Ho:YAG). The laser is absorbed significantly by water and hence can allow for the superficial cutting or ablation of water-rich tissue. Pulse modulation of lasers represents one of the most significant advances in laser technology for urological procedures. Moses™ technology (120 Hz holmium laser, Boston Scientific, Marlborough, MA, USA), first introduced in 2017, was developed to overcome potential limitations of traditional holmium laser [3]. The Moses™ effect describes a form of pulse modulation whereby the pulse is modified into two sub-pulses: an initial pulse generates a vapour bubble whilst the second passes through this bubble to its target tissue with greater energy [4,5]. The aim of pulse modulation is to increase the efficiency of energy transfer to the stone. This theory was first tested by Ibrahim et al. (2017) [6] on stone simulators and in a pre-clinical context where they found less retropulsion compared to conventional holmium, as well as lower energy lost through water [7]. Subsequent early in vitro studies by Yamashita et al. (2022) [8] also supported the enhanced performance where authors described statistically significant improvement in stone ablation efficiency as determined by stone volume/fragmentation. In the context of prostate enucleation, the European Association of Urology (EAU) guidelines support laser prostate enucleation as a size-independent treatment option for BPH and a potential replacement for transurethral resection of the prostate (TURP), which is associated with complications including infection and incontinence [9]. Despite many trials indicating the safety profile of holmium enucleation, there has not been widespread use of HoLEP across the UK - attributed in part to the steep learning curve. The use of Moses™ technology in prostate enucleation was first described by Large et al. (2020) [10], reporting more effective tissue ablation and separation and better formation of the surgical plane with subsequently enhanced haemostasis, all leading to a superior choice of laser modality for prostate enucleation.

Due to its optimised energy delivery, there is potential for Moses™ technology to enhance both laser lithotripsy and prostate enucleation. Given its relatively recent introduction, little is truly known of its beneficial impact and whether Moses™ offers an overall cost benefit. The aim of this review is to determine if Moses™ represents an efficacious option for laser lithotripsy and prostate enucleation, allowing endourologists to better plan for procedures.

## Review

Methods

Aims

The objective of this study was to compare the intra- and post-operative complications of Moses™ technology to standard holmium laser for the management of both urolithiasis and prostate enucleation.

Literature Search and Study Selection

A comprehensive literature search was conducted in the PubMed and Embase databases covering studies published from January 2016 to March 2024 to capture the most recent and relevant data on Moses™ technology and holmium laser use. The search strategy for stone lithotripsy included the following keywords and their combinations: "Lithotripsy", "Holmium Laser", "Moses", and "Pulse Modulation". For prostate enucleation, the search terms included "prostate hyperplasia", "Moses™", "pulse modulation" and "HoLEP". Additionally, reference lists of relevant articles were manually screened to capture any further eligible studies.

Two authors independently performed the screening and selection of studies in a two-step process: first by title and abstract and then through full-text review to assess eligibility. Discrepancies were resolved by discussion or consultation with a third reviewer.

Inclusion and Exclusion Criteria

Studies were eligible for inclusion if they involved patients with confirmed urinary stones or BPH and directly compared the use of Moses™ technology to conventional holmium:YAG laser techniques. For the urolithiasis portion of the review, studies were included if they involved laser lithotripsy using Moses™ or Moses™ 2.0 (120 W laser) and reported comparative outcomes between Moses™ mode and standard holmium laser. Key data such as operative time, lasing time, complication rates, stone-free rates, and retropulsion scores were required for inclusion.

Similarly, for the prostate enucleation component, studies were included if they evaluated intraoperative outcomes such as enucleation time, total operating time, haemostasis time, and total laser energy used, as well as postoperative complications and functional outcomes. These included metrics like catheterisation rates, hospital stay duration, urethral stricture incidence, and post-operative urinary flow measures.

Studies were excluded if they did not provide a direct comparison between Moses™ and standard holmium laser, or if they lacked sufficient data on relevant outcomes. Grey literature, such as case reports, case series, meeting abstracts, editorials, or correspondence was excluded due to limited methodological detail and lack of peer review. Non-English studies without accessible translations were also excluded to ensure consistency in interpretation and quality assessment.

Data Extraction and Outcome Measures

For stone lithotripsy studies, operative time was defined as the interval from initial ureteroscope insertion to final removal. Complications were primarily reported using the modified Clavien-Dindo classification. Stone-free status definitions varied but typically included no residual fragments >4 mm on computed tomography of the kidneys, ureters, and bladder (CT KUB) performed three to four months postoperatively. Retropulsion was assessed in some studies using Likert scoring (0=no retropulsion to 3=maximum retropulsion). Laser settings frequently used for fragmentation were 1 J and 10 Hz, and for dusting 0.4 J and 80 Hz.

For prostate enucleation, enucleation efficiency was calculated as grams of prostatic tissue removed per minute. Postoperative outcomes included length of hospital stay, re-catheterisation rates, urethral strictures, peak urinary flow rate and post-void residual volume.

Results

Moses™ in Lithotripsy

An initial search yielded 64 studies using the previously mentioned search criteria. A total of seven studies were included in the final analysis based on inclusion criteria: one was a randomised controlled trial, four were retrospective studies, one was prospective study and one a case-control study.

Table 1 shows a detailed summary of all studies, including outcomes. All studies compared the use of standard vs Moses™ laser and contained outcomes on safety and efficacy. Energy and frequency setting remained consistent for both dusting and fragmenting with low energy (0.3-0.4J), high frequency (60-80 Hz) for dusting, high energy (0.8-1.0 J), low frequency (8-10 Hz) for fragmenting.

**Table 1:** Study Characteristics and Outcomes for Laser lithotripsy using Holmium YAG laser in standard mode vs Moses mode * HU=Hounsfield units (a measure of stone density on CT imaging). ** Total duration of the procedure including access and retrieval. † Time spent actively fragmenting the stone using the laser. ‡ Duration the laser was activated during the procedure. § Proportion of patients with no residual fragments or clinically insignificant fragments (<4 mm) on follow-up imaging. RCT: Randomised controlled study; YAG: yttrium-aluminum-garnet.

Study	Study Design	Year	Total Patients (n)	Mean Stone Size (mm)	Mean Stone Density (HU)*	Operative Time (min)**	Fragmentation Time (min)†	Total Laser Energy (J)	Lasing Time (min)‡	Stone-Free Rate (%)§
Ibrahim et al. [6]	RCT	2020	36 vs 36	1.4 vs 1.7	841 vs 991	50.9 vs 41.1	21.1 vs 14.2	11.1 vs 10.8	7.4 vs 6.1	83.3 vs 88.4
Pietropaolo et al. [11]	Case-Control	2021	38 vs 38	1.18 vs 1.09	n/a	82.1 vs 51.6	n/a	n/a	n/a	97.3 vs 100
Wang et al. [12]	Retrospective Cohort	2022	102 vs 114	1.2 vs 1.2	994 vs 990	21.17 vs 18.39	n/a	n/a	5.94 vs 4.99	85.3 vs 86.8
Mullerad et al. [13]	Prospective	2017	11 vs 23	n/a	867 vs 901	n/a	n/a	6.4 vs 4.5	10.0 vs 6.0	n/a
Knoedler et al. [14]	Retrospective Cohort	2022	66 vs 110	1.16 vs 1.18	n/a	39.8 vs 43.5	17.1 vs 20.5	3.8 vs 5.1	6.7 vs 7.5	65.3 vs 62.3
Mekayten et al. [15]	Retrospective Cohort	2019	462 vs 169	n/a	1022 vs 1085	31.84 vs 21.13	n/a	3.6 vs 4.7	6.6 vs 3.3	87.2 vs 84.5
Majdalany et al. [16]	Retrospective Cohort	2021	18 vs 11	0.94 vs 0.94	784 vs 865	10.4 vs 14.3	n/a	6.4 vs 12.4	5.3 vs 7.0	71 vs 90

Operative Time

A total of six studies reported data on operating time. Of the studies, four reported statistically significant improvements in total operating time for Moses compared to standard holmium. In their prospective double blinded RCT in 2020, Ibrahim et al. (2020) [6] found mean operating time for Moses™ as 41.1 compared to 50.9 minutes for standard holmium (p=1.03). This was consistent with comparative studies by Pietropaolo et al. (2021) [11] who found mean Moses™ operating time to be 51.6 minutes compared to 82.1 minutes.

Stone-free Assessment

No studies reported statistically significant differences in stone-free rates, which ranged from 71% to 100% for both holmium standard and Moses.

Stone Fragmentation Time

Overall, two studies reported total stone fragmentation time: a randomised control trial by Ibrahim et al. (2020) [6] reported statistically significant reduction in total fragmentation time (21 minutes for standard holmium vs 14 minutes for Moses™ mode p<0.05); Wang et al. (2022) [12] found a statistically significant improvement in overall stone laser fragmentation time (4.99 minutes for Moses™ mode vs. 5.94 minutes for standard holmium, p<0.001) whilst Mekayten et al. (2019) found Moses™ mode demonstrated 234.91 seconds shorter fragmentation time controlling confounders such as stone volume, hydronephrosis, and location (p<0.0001) [15].

Stone Retropulsion

Ibrahim et al. (2020) [6] was the only study that formally assessed the degree of stone retropulsion based on Likert scoring. A statistically significant reduction in retropulsion rates was found with Moses. The authors stated it led to fewer pauses in fragmentation and therefore explained the reduction in overall procedure time.

Complication Rate

Overall complications related to ureteroscopy for both standard and Moses mode were similar. Ibrahim et al. (2020) [6] reported ureteric perforation intraoperatively based on retrograde ureterogram, which prompted ureteric stenting during the procedure. Subsequent ureteric stricture was found in the same patient.

Moses™ in Prostate Enucleation

A literature search initially identified 65 papers. After exclusions based on abstract and full text search, eventually five studies were incorporated into our analysis. Overall, two studies were randomised controlled trials, two were observational retrospective studies, and one prospective study. Table 2 shows a detailed summary of all studies, including outcomes.

**Table 2:** Characteristics and Outcomes for Prostate Enucleation Using Holmium YAG laser in Standard Mode vs Moses Mode * Three-lobe: Conventional anatomical enucleation; En bloc: Single-piece enucleation of the adenoma. ** Time spent achieving complete hemostasis using the laser. † Time required to complete adenoma dissection from the surgical capsule. ‡ Total procedure time from scope insertion to removal. § Reported in kilojoules (kJ); 1 kJ = 1,000 J. YAG: yttrium-aluminum-garnet; RCT: randomised controlled study.

Study	Study Design	Year	Enucleation Technique*	Total Patients (n)	Prostate Volume (g)	Hemostasis Time (min)**	Enucleation Time (min)†	Operative Time (min)‡	Total Laser Energy (kJ)§
Kavoussi et al. [17]	RCT	2021	Three-lobe	30 vs 30	153 vs 131	29 vs 19	80 vs 68	126 vs 101	143 vs 130
Nottingham et al. [18]	Retrospective Cohort	2021	Three-lobe	50 vs 54	n/a	10.6 vs 8.1	47.1 vs 45.9	91 vs 81	95.9 vs 110.4
Socarrás et al. [19]	Prospective	2023	En bloc	137 vs 80	75.5 vs 86.6	8.35 vs 3.01	31.46 vs 22.1	47.58 vs 32.16	125.86 vs 117.56
Large et al. [10]	Retrospective Cohort	2020	Three-lobe	217 vs 137	110 vs 155.6	10.6 vs 6.3	47.1 vs 40.9	n/a	n/a
Nevo et al. [20]	RCT	2020	Three-lobe	27 vs 27	n/a	6.5 vs 3.5	50.1 vs 45.4	n/a	n/a

Enucleation Time

Enucleation time was significantly shorter in all five studies in this analysis. Socarrás et al. (2023) [19] reported a shortened enucleation time by 29% (22.10 minutes for Moses and 31.46 minutes for standard holmium (p<0.001).

Haemostasis Time

Haemostasis time was found to be statistically shorter for Moses HoLEP compared to standard HoLEP in all five studies in this analysis.

Operative Time

This was analysed in a total of three studies. Socarrás et al. (2023) [19] found a 32% improvement in overall surgical time for Moses™ compared to the standard holmium laser for en bloc HoLEP (32.16 minutes vs 47.58 minutes).

Post-operative Complications

For short-term post-operative complications, there was a higher incidence of re-catheterisation and urethral stricture in standard HoLEP vs Moses™ although there was no statistical significance to these findings. Large et al. (2020) [10] and Nottingham et al.(2021) [18] reported post-void residual and q max. However, neither of these was found to be statistically significant differences; Nottingham et al. (2021) [18] also reported post-op International Prostate Symptom Score (IPSS) scores. However, these too weren’t significantly different.

Discussion

Moses™ and Stone Lithotripsy

The introduction of pulse modulation in 2017 represented a significant innovation in the field of endourology. Holmium laser currently is the gold standard modality for laser stone ablation. However, Moses technology and pulse modulation aimed to enhance energy delivery to the stone and ensure greater efficiency in stone ablation. Moses™ is itself a higher-powered modality of holmium YAG laser, working by the splitting of pulses into two sub-pulses. Typically, a direct relation between pulse energy and fragmentation rate exists, but also leads to a higher risk of retropulsion of stones and thus a potentially longer procedure time [6]. The first generation of Moses works within two pulse modulations: Moses contact at 1 mm from target and Moses distance at 2 mm. Early laboratory findings reported efficient ablation of soft stones in Moses contact mode [21]. Subsequently, clinical studies were conducted in various regions in recent years, and we aimed to synthesise and review these studies to determine the potential benefits Moses may have over conventional holmium laser.

In out narrative review, we found a total of seven studies comparing Moses™ technology to holmium laser in standard mode. A range of efficiency outcomes were reported, including operative and fragmentation time, lasing time and post-op complications. The study with the highest quality was a double-blinded randomised controlled trial by Ibrahim et al. (2020) [6] in 2020 highly favouring Moses to standard holmium with significantly better outcomes for operative time and fragmentation time - a 20 percent reduction in operating time, and a 33 percent reduction in fragmentation time was found. These findings were reinforced by later studies in 2021 by Pietropaolo et al. (2021) [11] and Wang et al. (2022) [12] and in 2022 both finding statistically significant reductions in overall operating time. Operative time is a key indicator of efficacy in this field; longer operating procedure can lead to higher risk of intra- and post-operative complications. Although only reported statistically within one study, retropulsion rates were favourable with Moses compared to holmium, thus enhancing the overall efficiency narrative. A reduced retropulsion will lead to a reduced overall operating time [6,12].

In terms of other outcome measures, regarding stone-free rate, a universally recognised indicator of procedure efficacy, we found no significant differences in studies for both laser modes. The definitions of stone-free status tended to vary in studies. Stone-free rates were ultimately stable for both modes in all papers included in this study. The safety profile of Moses™ has been reported by Winship et al. (2019) [22] whereby Moses™ produced overall less heat and reduced risk of ureteric injury. This was reinforced by Corsini et al. (2022) [23] where Moses™ was reported to have a lower impact on thermal injuries compared with holmium YAG lasers. Lower overall heat generation and reduction in thermal injury will lead to lower rate of ureteric stricture. We did not find significant differences in complication rates between both standard holmium and Moses™. However, all studies performed ureteroscopy at safe times, that is, no concurrent infection or conducted in patients with significant comorbidities where operating would be associated with significant risk. Moreover, all cases received appropriate preoperative management. Given the reduced operating time for Moses™, it may be suggested that the benefits of a shorter procedure will lead to better post op outcomes [15,16].

Overall laser usage time is a parameter utilised often in endourological studies and laser efficiency is typically defined as overall energy usage required to ablate a stone of size 1 mm [15]. Laser usage time benefits were found by Wang et al. (2022) [12] and Mullered et al. (2017) [13]. Wang et al. (2022) [12] did conclude enhanced efficiency defined as volume of stone ablated/laser working time, but ultimately this may be heavily dependent on the experience of the surgeon.

It is yet to be determined if the higher cost of Moses™ compared to standard holmium laser justifies widespread usage. Given the higher overall efficiency, it is suggested Moses™ may offer cost benefit over standard laser. However, few studies have formally investigated cost-benefit savings. In laser lithotripsy, Stern and Monga (2018) [4] reported that for stones of all sizes. Moses™ increased the cost by $292 per procedure despite reduced operating time. In the context of the National Health Service (NHS), no reports are available. However, the reduced overall operating time may increase capacity on lists whilst reducing waiting lists. Future studies should incorporate real-time cost savings in their analysis.

Moses™ 2.0

In 2020, next-generation Moses™ 2.0 technology was introduced. This now includes a modality known as optimised Moses™ where the option of extended frequency of 80-120 Hz is available. This is shown in lab studies to have better stone ablation than regular Moses™ distance mode. With the popcorn dusting technique whereby larger fragments are reduced in size to allow spontaneous passage, the distance between the laser fire tip and the stone is influential. Moses™ has two modes: contact for close distance and distant mode (1-2 mm). Black et al. (2025) [24] reported in theory that Moses™ may allow for better popcorn lithotripsy as photons will travel further through the initial pulse channel and when working at distance, the reduced motion of fragments may make it easier to keep fibre tip at the stone's surface. Black et al. (2025) [24] did find that Moses was superior in creating smaller fragments and subsequently reduced the risk of further intervention.

Moses 2.0 became the first holmium laser to provide frequencies greater than 100 Hz, claiming to have an enhanced ball-shaped tip to enable a smooth initial insertion of the fibre through flexible scopes, and a moving laser fibre to maximise the enhanced frequency. The Moses 200 D/F/L flexible fibre is reported to minimise scope deflection loss to allow for difficult-to-access stone locations. Recent laboratory studies with Moses 2.0 showed that by increasing fibre speed to 3 mm/s in Moses Distance mode, these enhanced overall ablation speeds significantly. It was also reported in early studies to be more operator-friendly. The Moses system had dual pedal footswitches for the surgeon, easily alternating between laser settings without requiring the surgeon to change laser settings on the laser console. The use of Moses 2.0 is more expensive however (approximately a $119 premium to standard). No current studies report cost-benefit analysis of the use of this newer technology in a UK-based setting and ultimately there are very few studies reporting clinical experiences of Moses 2.0 mode compared to Moses 1.0 in general [24].

Moses™ and Prostate Enucleation

It is hypothesised that energy and efficiency benefits for Moses™ in lithotripsy could be translated to similar benefits in prostate enucleation. In reality, adoption of HoLEP has been slow due to intraoperative difficulties, including the finding of correct plane, scarring of tissue and achieving adequate haemostasis. If Moses™ does indeed reduce these parameters, then it could shorten the learning curve for laser prostate enucleation globally. In our narrative review, we found papers reporting beneficial outcomes for Moses™ HoLEP in terms of enucleation time, haemostasis time and in turn operative time, suggesting that Moses is, in fact, superior to standard holmium for enucleation.

The first randomised controlled trial comparing Moses™ and holmium was conducted by Kavoussi et al. (2021) [17] in 2021 and found significantly reduced total operating time, haemostasis time and enucleation time for men older than 45 with a prostate size greater than 80 cc and moderate lower urinary tract symptoms (LUTS) as per IPSS score. Using multivariate analysis, they found 3.9 times reduction in operating time compared to HoLEP. The authors attributed this to improved energy transfer and enhanced visibility. Furthermore, Nottingham et al. (2021) [18] attributed their findings of lower haemostasis time to the speed of controlling tissue haemorrhage, clearing surgical field, enhanced visualisation of the prostatic capsule and easier development of the correct plane - factors which might lead to a shallower learning curve for laser prostate enucleation.

Haemostatic benefits were also reported by Large et al. (2020) [10] who found a lower reduction in postoperative haemoglobin levels in the Moses™ group, which was statistically significant, increasingly relevant with rising use of anticoagulants in patients.

Better energy delivery leads to more efficient ablation and improved visibility, making it easier for the surgeon. This can explain reduced operating time. In the paper by Nottingham et al. (2021) [18], the authors stated that Moses™ mode was able to achieve an optimum balance between cutting and coagulation: typically use of holmium involves high peak power to facilitate optimum dissection between capsule and adenoma and this can compromise coagulation efficacy. Furthermore, Whiles et al. (2019) [25] reported time savings of 12.3 minutes for the 50 g prostate in the operating room.

In terms of post-operative complications, studies reported rates of incidence of re-catheterisation, and urethral stricture, reporting higher rates for both in HoLEP groups than for Moses™. Some studies also reported the same for post-operative functional parameters incorporated in determining a successful enucleation of the prostate including Qmax and mean post-void residual volumes, both of which were reported as lower in two studies (Large et al. (2020) [10] and Nottingham et al. (2021) [18]) for the Moses™ group. However, these were not found to be statistically significant in either and the actual effect of Moses™ on these is difficult to interpret [9,18].

There is a universal aim to facilitate same-day discharge for HoLEP patients. This aim was reported in Nottingham et al. (2021) [18], who observed a reduced degree of haemostasis with minimal to no haematuria post-operatively, overcoming the biggest obstacle to same-day discharge, whilst reducing need for continuous bladder irrigation to prevent clot retention - 70 percent of their cohort was sent home on the same day with their catheter out. These findings support the view that HoLEP can be performed as an outpatient-led procedure.

Laser fibre degradation is an issue in HoLEP as it can lead to scope damage and the need to remove the fibre for stripping, leading to prolonged operation time. Moses™ has been seen to be associated with lower fibre degradation. This is perhaps due to the formation of the cavitation bubble. Nevo et al. (2021) [20] report lower fibre degradation in Moses™ - mean of 3.5 nm, significantly less than 16.8 nm with holmium. Two papers in this study (Large et al. (2020) [10] and Nottingham et al. (2021) [18]) report similarities with reduced fibre degradation. A study by Assmus et al. (2022) [26] investigated fibre tip degradation of Moses™ vs regular holmium by quantitative measurement of pre- and post-operative laser tip length, finding Moses™ to be superior. The reduction in fibre tip degradation reduced overall energy output, and the need for repeat stripping prolonging the operative time. Nevo et al. (2021) [20] investigated extensively a comparison of intraoperative performance of Moses™ including parameters such as incision sharpness, fibre control, tissue separation, visibility and charring - all subjectively evaluated by reviewers via video post-procedure. They concluded that Moses™ had better tissue separation from the expanding steam bubble and effective dissection from enhanced exposure to the correct planes, superior to standard holmium, all supporting the view that Moses is superior to standard holmium.

Limitations

Ultimately, all the reported studies have some limitation. It becomes a challenging task to compare outcomes in endourological procedures due to differences in the skill of the surgeon, settings for lithotripsy study types and locations of stones, size and density of stones, leading to significant heterogeneity. Unfortunately, no studies incorporated stone composition analysis into their data on operative time and fragmentation efficiency. We know different types of stones are associated with differing rates of fragmentation given that men have a higher probability of calcium oxalate formation, whilst women have infection stones with looser structures.

Our most significant findings were benefits in operating time. Yet, the total operating time is not an ideal parameter as it can be influenced by issues with initial scope insertion, need for urethral dilation, or even malfunctioning equipment and time taken to troubleshoot/source equipment within the procedure.

Moreover, the number of available studies, particularly regarding Moses™ 2.0, remains limited. As such, the findings related to this newer technology should be interpreted as preliminary and hypothesis-generating. Further high-quality, comparative studies are needed to validate its purported advantages.

A key limitation in the current literature is the lack of sub-group analysis comparing Ho:YAG and Moses technology based on stone size and location. As the difficulty of stone treatment can vary greatly depending on these factors, the absence of this data makes it challenging to draw definitive conclusions regarding the superiority of Moses technology.

Another important consideration is the potential risk of bias within the included studies. Due to the narrative review format, the formal risk of bias assessments were not conducted. However, heterogeneity in study designs, patient populations, and outcome measures introduces variability that could influence the interpretation of results. This should be taken into account when considering the overall conclusions.

Furthermore, there have been recent developments in thulium laser, showing improved stone-free rates and fewer complications compared to holmium. Thulium is shown to have a higher water absorption peak and thus lower threshold for ablation [4]. So further studies comparing thulium to holmium Moses would be beneficial.

## Conclusions

Moses™ technology represents a notable advancement in laser lithotripsy, demonstrating consistent improvements in operative efficiency. Across the reviewed studies, Moses™ was associated with reductions in total operative time, lasing time, fragmentation time, and energy usage compared to standard holmium lasers. While stone-free rates remained comparable, reduced retropulsion and shorter procedures suggest potential for better patient outcomes and increased procedural throughput.

For prostate enucleation, the evidence more clearly supports the superiority of Moses™, with consistent findings of reduced enucleation and haemostasis times, improved intraoperative visibility, and potential to shorten the learning curve for HoLEP. These features may support broader adoption of the procedure and offer feasibility for outpatient treatment. However, cost-effectiveness remains under-investigated and requires further study, particularly in NHS settings. Future comparisons with newer modalities such as thulium lasers will be important to determine the optimal tool for endourological procedures.
